# Efficacy of nanosecond laser treatment in central serous chorioretinopathy with and without atrophy of retinal pigment epithelium

**DOI:** 10.1186/s40942-020-00214-3

**Published:** 2020-06-04

**Authors:** Hakan Kaymak, Saskia Funk, Andreas Fricke, Roxana Fulga, Karsten Klabe, Berthold Seitz, Achim Langenbucher, Hartmut Schwahn

**Affiliations:** 1 Internationale Innovative Ophthalmochirurgie, Theo-Champion-Str. 1, 40549 Duesseldorf, Germany; 2 Department of Ophthalmology, Saarland University Medical Center, Kirrberger Str. 100/22, 66424 Homburg, Germany; 3 Institute of Experimental Ophthalmology, Saarland University Medical Center, Kirrberger Str. 100/22, 66424 Homburg, Germany

**Keywords:** Central serous chorioretinopathy, CCS, CSC, CSCR, Microperimetry, Retinal pigment epithelium, Subretinal fluid, Subthreshold laser, Nanosecond laser

## Abstract

**Background:**

To evaluate the outcomes of subthreshold nanosecond laser treatment of chronic central serous chorioretinopathy (CSC) as a function of the severity of concomitant of retinal pigment epithelium (RPE) defects.

**Methods:**

This retrospective study compares data from 23 CSC diagnosed eyes with only mild RPE defects (group 1), 16 CSC eyes with moderate RPE defects (group 2), and 17 CSC eyes having severe RPE defects (group 3). After subthreshold treatment with the standard Ellex 2RT™ nanosecond laser (Ellex Medical Lasers Ltd, Australia), changes in macular structure and levels of subretinal fluid (SRF) were assessed by OCT-SD, OCT-A, functional integrity of the retina was assessed by corrected distance visual acuity (CDVA) and microperimetry, each at baseline and 1, 3, 6, and 12 months after initial treatment; re-treatment took place in cases of persistent SRF *pro re nata*.

**Results:**

During the 12 months observation period, group 1 and 2 mostly required on initial and one re-treatment (1.9 ± 1.0 treatments; 1.9 ± 1.3 treatments). In contrast, group 3 was subject to three to four treatments (3.7 ± 1.5 treatments). 6 to 12 months after treatment, subretinal fluid (SRF) disappeared in 100% of the eyes of group 1 and in 76.9%, and 42.9% of the eyes of group 2 and group 3, respectively. Retinal sensitivity and CDVA improved in group 1 and 2, but did not change significantly in group 3 during the 12 months period.

**Conclusions:**

Subthreshold nanosecond laser treatment is an effective and safe method for the restoration of macular anatomy and sensitivity in acute and chronic CSC cases with only mild or moderate RPE defects. However, this laser treatment has very limited outcome in CSC eyes with more severe RPE defects.

## Background

Central serous chorioretinopathy (CSC) is a chorioretinal pathology mostly affecting the middle-aged population. It is an atypical form of macular edema with accumulation of fluid under the retina, in the subretinal space, i.e. subretinal fluid (SRF) [1, 2], mostly due to retinal pigment epithelium (RPE) barrier breakdown [1]. There is spontaneous recovery from acute CSC with a complete absorption of SRF in most patients. However, there is still a significant number, i.e. around 30%, of CSC patients who suffer from chronic and recurrent episodes, which in some cases results in considerable visual impairment [2]. Accordingly, CSC can be classified as acute, chronic, or recurrent, although classification of cases is not consistent among authors [3]. Some factors have been reported to correlate positively with the chronic form of the disease, such as advanced age at onset, duration of the disease [4]. Likewise, some factors have been generally identified as risk factors for CSC, such as steroids, antidepressant or anxiolytic drugs, smoking, pregnancy and hyperopia, whereas myopia has been defined as a protective factor against CSC [4].

The criteria for selecting the most appropriate therapeutic option and also the long-term outlooks of each treatment option remain under discussion, especially for chronic and more complex forms of CSC [5, 6]. The application of a subthreshold laser therapy for acute and chronic CSC has been previously validated [6–16]. There is also scientific evidence that supports the use of subthreshold laser therapy in eyes with chronic CSC and associated RPE defects [17]. However, it remains unclear whether there are differences in the therapeutic effect of subthreshold laser treatment with respect to the level of severity of RPE defects. Treatment options that have been widely studied for the last years include laser photocoagulation directed at extrafoveal focal retinal pigment epithelium (RPE) leaks, subthreshold laser treatment in eyes with leakage sites within or very close to the foveal avascular zone, and Verteporfin (Visudyne^®^) based photodynamic therapy (PDT) directed at choroidal hyperpermeability areas [6–12, 18, 19]. By mild non-lesioning laser stimulation of retinal pigment epithelium (RPE) underlying the retina, several regeneration processes and increased metabolism and transport mechanisms take place and RPE proliferation is stimulated. These effects are thought to lead to a re-constitution of the blood-retinal barrier in the CSC affected retinal area and is expected to support SRF resorption and thus recovery from CSC.

In common PDT, the closure of choriocapillaris and ischemia induced by PDT may generate oxidative stress in the RPE and may thus even aggravate the epitheliopathy underlying the CSC [13, 20]. Micropulse laser treatment utilizes a series of short laser pulses and thus allows better control over the laser energy delivered to the anterior segment of the eye and thus may protect against potential adverse thermal effects to tissue surrounding the RPE. In fact, micropulse laser therapy has been reported to allow treatment of subfoveal and juxtafoveal leaks without inducing visible laser burns and thus avoiding some adverse events known to be associated with PDT, including transient reduction of macular function, choroidal non-perfusion, RPE atrophy and choroidal neovascularization (CNV) [6, 8, 10, 11, 21].

In the meantime, the advent of the micropulse laser has triggered the idea to develop a nanosecond laser (or “nanolaser”) delivering ultrashort pulses of laser energy of only about 0.1 mJ to 0.5 mJ. By such ultrashort pulses, the target tissue, the RPE, can be stimulated even more effectively and without any considerable risk of thermal injury to the surrounding tissue. There are reports on favorable outcome of nanosecond laser therapy on diabetic macular edema see, for example, [22].

In spite of the potential benefits of such subthreshold laser treatment of CSC, it is still unknown if such treatment is applicable to all forms of CSC with reasonable expectation of success. In particular, the presence of RPE defects, which can be commonly found in chronic and severe forms of CSC [23], may limit the results of this therapy. The rationale behind this considers the RPE as the major target of the subthreshold laser treatment and a stimulated functional RPE to effect the reduction of the symptoms of CSC, specifically the presence or amount of SRF [24]. Where there is remaining functional RPE, subthreshold laser stimulation of that functional RPE may trigger the drainage of SRF and thus reduce CSC. But when the RPE is considerably damaged, such damaged RPE might no longer support SRF drainage even after (repeated) subthreshold laser stimulation.

The aim of this study was to evaluate the outcomes of a CSC therapy by subthreshold nanosecond laser stimulation as a function of the level of RPE defects according to the publication of Lee et al. [25].

## Methods

### Patients and classification

**Fig. 1:**
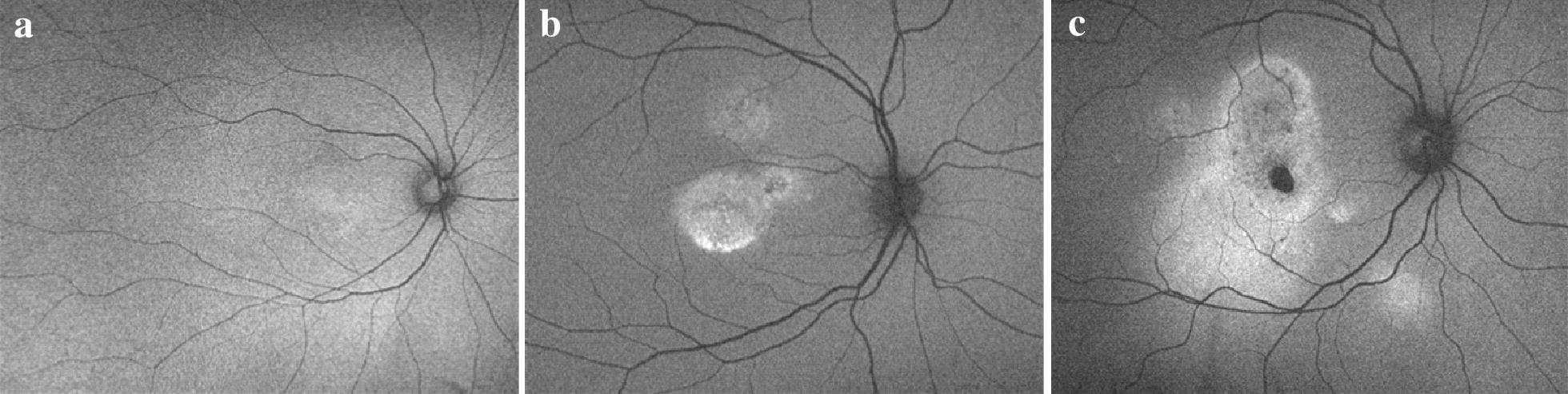
Comparison of the RPE defects in the autofluorescence images of the three groups **a** group 1 mild RPE defects, **b** group 2 with moderate RPE defects, **c** group 3 with severe RPE defects

This observational, single-center retrospective study includes data from 56 eyes of 55 patients with an age range of 27 to 56 years. All patients underwent our standard care treatment for CSC patients, who were not eligible for standard PDT-treatment approved by the public health insurance system. In this study, data was taken from patients which had a diagnosis of CSC, defined as the association of visual symptoms (vision impairment, metamorphopsia, micropsia, dyschromatopsia or central scotoma) and a presence of subretinal fluid (SRF) as determined by OCT data. Data from patients, which have had CNV as evidenced by fluorescein angiography or had refractive errors of more than 2 D or had previous medical records or CSC-related symptoms as detected in fundus examination that would have suggested any recurrent SRD had undergone previous CSC laser treatment or suffered from any other active ocular condition like glaucoma or macula disease, was not used in this study. Eligible patient data was splitted into three groups (Fig. 1) according to the severity level of the RPE defects. The individual severity levels of the defects were evaluated independently by three masked retina specialists in accordance with the criteria established by Lee and colleagues [25], mainly based on SD-OCT, fundus autofluorescence imaging (FAF), and OCT-A: Group 1 (n = 23) contains data from patients with CSC symptoms like visual loss or metamorphopsia observed for more than 3 months but for less than 8 months and with only mild changes in RPE or suspicion of symptom recurrency; in the area of serous retinal detachment (SRD), homogeneous hyperautofluorescence (Hyper-FAF) is higher than the FAF background intensity in peripheral retinal areas. Group 2 (n = 16) is characterized by similar findings, but patient had more significant, yet moderate RPE defects and a history of CSC symptoms of more than 8 months. In this group, hyper-FAF is frequently observed in the area of SRD, and FAF is more heterogeneous, i.e. small dots with increased FAF intensity are displayed. Data eligible for group 3 (n = 17) originated from patients that had a history of symptoms of more than 24 months and significant and major RPE defects. In this group, an absolute focal hypo-FAF is present and a mixed pattern of hyper- and hypo-FAF can be observed over areas of RPE atrophic thinning.

### Examination protocol

Data are from patients which all underwent a complete ophthalmological examination under current standards of health care and good clinical practice including clinical history. Angio-optical coherence tomography was performed with the OCT (Cirrus HD 5000, Zeiss Meditec, Jena, Germany), horizontal scans through the fovea in enhanced-depth imaging (EDI) mode were acquired. Fluorescein and indocyanine green angiography (Visucam 500, Zeiss Meditec, Jena, Germany) were performed at baseline and repeated if needed, based on clinical examination and the results of OCT analysis. Fundus and fundus autofluorescence images were acquired with the Ultra Wide Angle Cameras Daytona or California (Optos Inc., Marlborough, MA, USA). For functional assessment, confocal microperimetry was performed with the MAIA (Centervue SpA, Padua, Italy) at 10° centre with 37 stimuli (“standard macular test”) and 4-2 strategy as described in detail by Dolar-Szczasny et al. [26].

Central foveal thickness was assessed automatically with the Zeiss Cirrus OCT system. Subretinal fluid (SRF) depth and choroidal thickness based on calibrated Cirrus HD-OCT software were performed by the same experienced technician to avoid variation in analysis. Measurements were taken perpendicular to the fovea from RPE basal membrane RPE to the border line visible between the choroid and the sclera on. The choroidal vascularity index is independent of systematic and ocular factors, whereas the choroidal thickness may be affected by physiological parameters [27, 28].

### Laser treatment

All patients underwent laser therapy utilizing the Ellex 2RT™ nanosecond laser (Ellex Medical Lasers Ltd, Adelaide, Australia) under topical anaesthesia. The Ellex 2RT™ nanosecond laser device is a commercially widely available Q-switched, green Nd:YAG laser (532 nm) with a pulse duration of 3 ns and a spot size of 400 μm with a speckled-beam profile as described in [22, 29–31]. A grid pattern was used to apply the laser spots, inside the area of serous detachment as previously determined in the fundus imaging, as well as in the area surrounding the detachment. An average energy of 0.17 mJ per each spot was observed. This laser energy level was selected on the basis of published reports where SRT and nanolaser had already been used to treat retinal conditions such as macular edema or age related macular degeneration [24, 31–34]. In the actual treatments, eyes of group 1 received 70 ± 17 spots at 0.14 ± 0.0.4 mJ/spot; eyes of group 2 received 72 ± 15 spots at 0.18 ± 0.04 mJ/spot, and eyes group 3 received 61 ± 18 spots at 0.18 ± 0.04 mJ/spot; there was no significant intergroup difference in the treatment.

### Statistical analysis

**Fig. 2:**
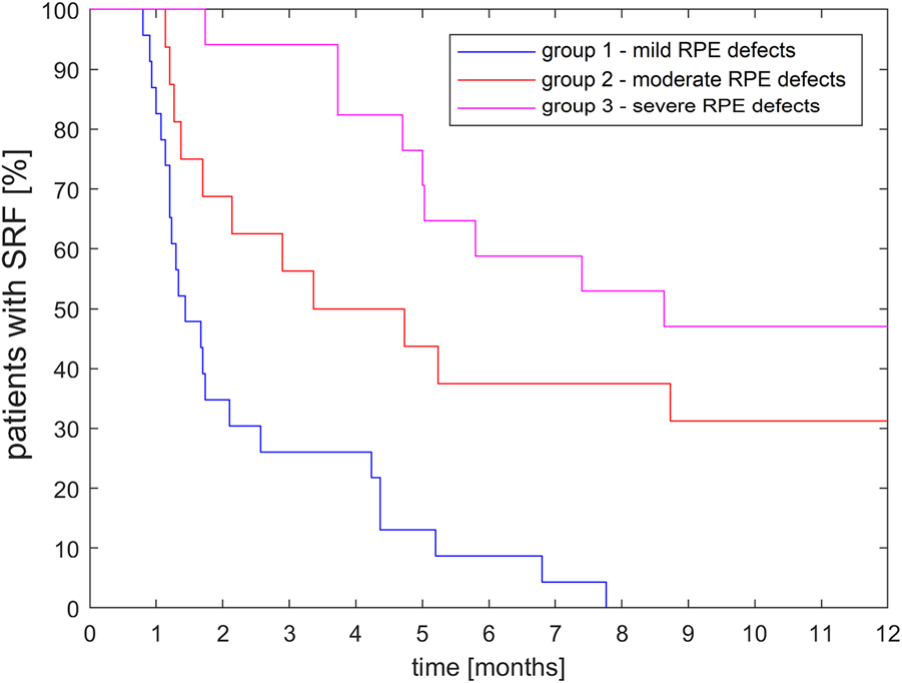
Kaplan–Meier survival analysis of the three groups. Patients are shown without subretinal fluid if they did not have any recurrence during the observation time

In order to assess possible multiple differences between and within the classified CSC groups we performed a Holm-Bonferroni test (OriginPro 2017 SR1, OriginLab Corp., Northampton MA, USA). To evaluate the level of SRF absorption, a Kaplan–Meier survival analysis (Fig. 2) was performed with a Logrank test in MATLAB (R2017b, Mathworks, Natick, USA).

## Results

All three groups had a high prevalence of males (group 1: 91.3%; group 2: 81.3%; group 3: 94.1%). Mean age did not differ significantly between the groups (group 1: group 2: 44 ± 9 years; 50 ± 11 years; group 3: 44 ± 11 years).

In the event persistent SRF was found, based on physician’s individual decision, patients received one or more additional laser treatment: In group 1, 10 (43%) patients received one re-treatment 40 ± 20 days after the initial treatment, resulting in a group average of 1.9 ± 1.0 treatments. In group 2, 3 (19%) patients received one re-treatment 69 ± 23 days after the initial treatment, resulting in a group average of 1.9 ± 1.3 treatments. In group 3, 10 (59%) patients received re-treatment up to 6 times 42 ± 1.5 days after the initial treatment, resulting in a group average of 3.7 ± 1.5 treatments.

**Fig. 3:**
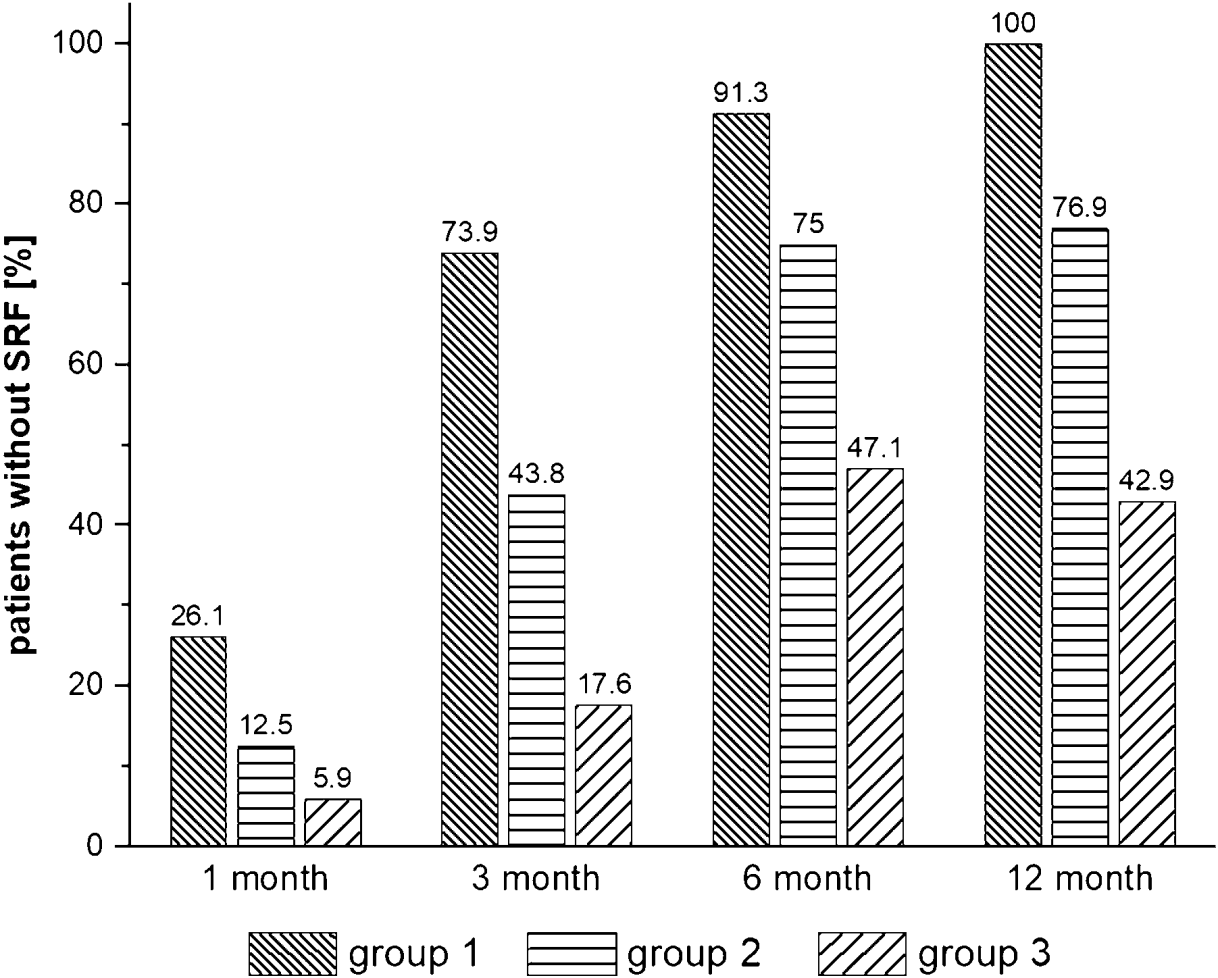
Distribution of cases with resolution of subretinal fluid in the three groups of eyes

Figure 3 shows the percentages of cases with full SRF absorption in the 12 months observation period: Latest at 12 months after initial treatment, group 1 had complete recovery from SRF, but only 76.9% of the eyes of group 2. Only 42.9% of the eyes of group 3 had completely resolved SRF during the 12 months period. The percentage of recurrence was 4.3% (i.e. one out of 23) in group 1, 12.5% (two out of 16) in group 2, and 23.5% (four out of 17) in group 3, respectively, during the 12 month observation period.

Table 1 summarizes the main clinical outcome measures obtained during the follow-up: Already after 1 month, a significant reduction in SRF and in central foveal thickness was observed in group 1 and group 2. An improvement in CDVA was observed at 12 months after treatment in group 1. There is a significant difference in the CDVA at baseline between group 1 and group 3 as well as between group 2 and group 3. There was no difference between group 1 and group 2 (Additional file 1: Table S1).

**Table 1:** Main visual acuity, central foveal thickness and high of subretinal fluid in classified eyes CSC eyes

	Group 1	Group 2	Group 3
Visual acuity (log MAR)			
Baseline	0.18 ± 0.17 (-)	0.23 ± 0.19 (-)	0.36 ± 0.34 (-)
1 month	0.17 ± 0.19 (0.90)	0.22 ± 0.16 (0.86)	0.35 ± 0.34 (0.93)
3 month	0.09 ± 0.18 (0.14)	0.20 ± 0.24 (0.70)	0.32 ± 0.32 (0.72)
6 month	0.11 ± 0.16 (0.30)	0.14 ± 0.13 (0.21)	0.40 ± 0.24 (0.80)
2 month	0.09 ± 0.14 (0.03)	0.16 ± 0.15 (0.11)	0.39 ± 0.35 (0.65)
Central foveal thickness (µm)			
Baseline	438 ± 189 (-)	366 ± 101 (-)	338 ± 147 (-)
1 month	280 ± 81 (< 0.01)	288 ± 55 (< 0.01)	286 ± 92 (0.18)
3 month	265 ± 59 (< 0.01)	269 ± 60 (< 0.01)	305 ± 106 (0.42)
6 month	280 ± 87 (< 0.01)	251 ± 63 (< 0.01)	297 ± 97 (0.32)
12 month	245 ± 31 (< 0.01)	253 ± 59 (< 0.01)	286 ± 80 (0.11)
Subretinal fluid (µm)			
Baseline	208 ± 198 (-)	140 ± 84 (-)	139 ± 130 (-)
1 month	48 ± 81 (< 0.01)	53 ± 51 (< 0.01)	91 ± 80 (0.01)
3 month	21 ± 50 (< 0.01)	34 ± 46 (< 0.01)	102 ± 96 (0.01)
6 month	38 ± 89 (< 0.01)	19 ± 49 (< 0.01)	82 ± 98 (< 0.01)
12 month	0 ± 0 (< 0.01)	18 ± 44 (< 0.01)	60 ± 74 (< 0.01)

A decrease in choroidal thickness of approx. 30 µm was observed in all three groups after 12 months after first treatment, interestingly the untreated fellow eyes had an average decrease of ca. 10 µm in the same respective observation periods.

Macular integrity and average threshold of microperimetry significantly increased in group 1 and group 2 already at first month after treatment and during the 12 months observation period (Table 2). In particular in group 1, macular integrity as assessed by microperimetry improved from “abnormal” (83.5 ± 28.7 O) before the treatment to “normal” (28.5 ± 30.8%) and mean average threshold of microperimetry improved from “suspect” (24.1 ± 4.1 dB) to “normal” (28.7 ± 3.2 dB). In group 2, macular integrity increased from “abnormal” (84.5 ± 32.2%) to “suspect” (49.2 ± 43.4%), and mean average threshold increased from “suspect” (24.3 ± 2.9 dB) to “normal” (27.8 ± 2.1 dB). In group 2, improvement in microperimetry functional parameters was some less than in group 1. Nevertheless, the differences in the outcomes between group 1 and 2 were not significant. In group 3, however, no significant improvement in these functional parameters could be observed: Macular integrity and mean average threshold of microperimetry remained in the “abnormal” range over the entire 12 months observation period. Accordingly, the positive outcomes of subthreshold laser treatment in groups 1 and 2 were each statistically significant from the outcomes in group 3 (Additional file 1: Table S2).

**Table 2:** Microperimetry parameter-macular integrity and average threshold for CSC eyes in classified groups

	Group 1	Group 2	Group 3
Macular integrity (%)^*^			
Baseline	83.5 ± 28.7 (-)	84.5 ± 32.2 (-)	84.1 ± 31.1 (-)
1 month	58.4 ± 38.7 (0.03)	78.6 ± 23.6 (0.69)	94.0 ± 8.0 (0.42)
3 month	39.1 ± 37.9 (< 0.01)	71.6 ± 26.3 (0.38)	84.6 ± 21.2 (0.96)
6 month	28.9 ± 37.1 (< 0.01)	50.8 ± 27.5 (0.06)	87.8 ± 28.1 (0.76)
12 month	28.5 ± 30.8 (< 0.01)	49.2 ± 43.4 (< 0.05)	81.6 ± 32.0 (0.84)
Average threshold (dB)^+^			
Baseline	24.1 ± 4.1 (-)	24.3 ± 2.9 (-)	20.1 ± 7.1 (-)
1 month	26.6 ± 3.4 (0.03)	25.9 ± 3.0 (0.20)	23.2 ± 2.4 (0.14)
3 month	28.3 ± 2.7 (< 0.01)	26.1 ± 2.0 (0.13)	25.1 ± 2.2 (0.02)
6 month	28.5 ± 3.8 (< 0.01)	28.1 ± 0.4 (0.01)	22.9 ± 3.9 (0.18)
12 month	28.7 ± 3.2 (< 0.01)	27.8 ± 2.1 (0.02)	23.9 ± 4.3 (0.06)

^*^Categories macular integrity: normal 0–40; suspect 40–60; abnormal 60–100

^+^Categories average threshold: normal 36–26; suspect 26–24; abnormal 24–0

## Discussion

The aim was to evaluate the outcomes of nanosecond laser treatment of CSC with a commercially available standard nanosecond laser system, here: the Ellex 2RT™ System as an example, and to determine how the therapeutic outcome depends on the level of severity of the concomitant RPE defects as classified in minor, mild, and severe RPE defects.

By means of subthreshold nanosecond laser treatment (average energy of 0.17 mJ per spot), SRF was absorbed in all the CSC patients which had only little or no RPE visible defects (group 1) latest at 12 months after treatment. This confirms the efficacy of this new therapeutic option in such a group of patients. As expected, nanosecond laser treatment had an excellent safety profile, as no CNV or RPE atrophy were observed during the follow-up. A significant reduction in SRF volume was observed already during the first 3 months. This is consistent with previous studies reporting the applicability of subthreshold laser treatment in acute CSC [7, 35, 36] for providing a faster visual rehabilitation than the standard of care (observation).

Our findings are generally consistent with previous clinical studies evaluating the outcomes of subthreshold laser treatment in chronic CSC [9, 10, 13–15, 17]. Arsan and colleagues [15] found reductions in central macular thickness and central macular volume in chronic CSC by micropulse laser treatment. Khatri et al. [17] evaluating the outcomes of micropulse laser treatment in chronic CSC, reported a change in central macular thickness and macular volume. In agreement with the significant reduction in SRF, a significant reduction of macular volume was also observed in the group having only minor RPE defects (group 1 and group 2). Therefore, the presently used standard 2RT™ nanosecond laser is a viable therapeutic option for effective treatment of CSC in eyes which show only minor RPE atrophies.

We also evaluated the outcomes of this subthreshold nanosecond laser treatment in eyes with already show severe RPE defects or abnormalities (group 3). In these eyes, the positive results of subthreshold nanosecond laser treatment could not be attained to the same level. In fact, for these group of eyes there was no significant improvement in macular volume or SRF resorption, and consistently no functional improvement in as assessed by CDVA and microperimetry. Only slight improvement was observed, but all well below statistical significance, even after multiple laser treatments during the 12 months period. Eventually, only 42.9% of the eyes of group 3 exhibited a complete resorption of SRF which is close to the rate reported for spontaneous recovery. Our findings confirm that the presence of more severe RPE defects in CSC is a limiting factor for the outcome of nanosecond laser treatment. The data of the present study reveals that the level of functional improvement in fluid resorption from the subretinal space by nanosecond laser stimulation significantly depends on the remaining level of RPE health and functionality. This suggests that SRF is efficiently resorbed only when there is sufficient intact RPE remaining.

There is reported superiority of half-dose PDT over subthreshold laser treatment [13, 37]. Van Dijk et al. [25] confirmed in a randomized controlled clinical trial that half-dose PDT led to a significantly higher proportion of complete resorption of SRF and functional improvement than subthreshold laser treatment due to the capability of this technique of inducing a significant decrease of choroidal perfusion [8]. However, half-dose PDT has been associated with potential complications, such as choroidal neovascularization [6], that were not found in our series treated with the nanosecond laser. Thus for the decision whether or not to choose subthreshold laser therapy or to choose PDT for a particular patient, the remaining integrity and functionality of the RPE should be individually assessed before treatment. For already severe RPE defects subthreshold nanosecond laser therapy may not prove effective, and alternative treatments like half-dose PDT should be given preference despite of their potential higher risk of complications or it should be considered not to start treatment in these eyes at all to minimize the risk over any potential benefit. For eyes with only mild RPE defects and, in particular, at early chronic CSC, however, this study evidences significant benefits of subthreshold nanosecond laser therapy. In cases of more severe RPE atrophy, a subthreshold nanosecond laser therapy can be applied, but half-dose PDT may be necessary in addition during the follow-up as the second line treatment.

The present results may justify a change in the existing reimbursement restrictions of (public) health insurance coverage in several countries regarding the off-label use of PDT in treating CSC. The treatment with the nanosecond laser could be good alternative therapy until approval of PDT.

## Conclusion

Subthreshold nanosecond laser therapy is a viable option for effective and safe restoration of macular anatomy and functional sensitivity in CSC where there are only minor RPE defects or abnormalities. Subthreshold nanosecond laser therapy has a much more limited outcome in eyes with more severe RPE defects. As a practical advice, the greater the damage to the RPE, the lower the therapeutic effect expected.

## Supplementary information

**Additional file 1: Table S1.** Statistic from Table 1—intergroup comparison. **Table S2.** Statistic of Table 2—intergroup comparison.

## Data Availability

The dataset used and/or analyzed during the current study are available from the corresponding author on reasonable request.

## Abbreviations

CDVA: Corrected distance visual acuity
CNV: Choroidal neovascularization
CSC: Central serous chorioretinopathy
OCT: Optical coherence tomography
FAF: Fundus autofluorescence
PDT: Photodynamic therapy
RPE: Retinal pigment epithelium
SRF: Subretinal fluid
SRD: Serous retinal detachment
