# Supplementary material for: Efficacy of nanosecond laser treatment in central serous chorioretinopathy with and without atrophy of retinal pigment epithelium
Source: Int J Retina Vitreous. 2020 Jun 4;6:11. doi: 10.1186/s40942-020-00214-3 (PMC7271527; doi:10.1186/s40942-020-00214-3)
Supplement: Supplementary file 1 — Additional file 1: Table S1. Statistic from Table 1—intergroup comparison. Table S2. Statistic of Table 2—intergroup comparison. [file 40942_2020_214_MOESM1_ESM.docx]

**Additional file 1**

**Tables**

**Table S1:** statistic from table 1 – intergroup comparison

|  | **Group 1 vs. 2** | **Group 1 vs. 3** | **Group 2 vs. 3** |
| --- | --- | --- | --- |
|  | p-value | p-value | p-value |
| **Visual acuity (logMAR)** Baseline 1 month 3 month 6 month 12 month | 0.50 0.55 0.22 0.80 0.60 | 0.02 0.02 0.01 <0.01 0.01 | 0.03 0.12 0.22 0.01 0.03 |
| **Central foveal thickness (µm)** Baseline 1 month 3 month 6 month 12 month | 0.16 0.76 0.89 0.44 0.14 | 0.05 0.79 0.14 0.62 0.89 | 0.60 0.95 0.23 0.20 0.16 |
| **Subretinal Fluid (µm)** Baseline 1 month 3 month 6 month 12 month | 0.18 0.83 0.56 0.57 0.92 | 0.17 0.07 <0.01 0.18 0.06 | 0.98 0.14 0.01 0.06 0.07 |

**Table S2:** statistic of table 2 - – intergroup comparison

|  | **Group 1 vs. 2** | **Group 1 vs. 3** | **Group 2 vs. 3** |
| --- | --- | --- | --- |
|  | p-value | p-value | p-value |
| **Macular Integrity** Baseline 1 month 3 month 6 month 12 month | 0.94 0.11 0.02 0.23 0.27 | 0.97 0.007 0.003 <0.01 0.001 | 0.97 0.29 0.40 0.05 0.09 |
| **Average Threshold** Baseline 1 month 3 month 6 month 12 month | 0.90 0.53 0.05 0.86 0.67 | 0.050 0.009 0.005 0.003 0.005 | 0.08 0.08 0.36 0.01 0.03 |
